# Echinococcosis/Hydatidosis in Ilam Province, Western Iran

**Published:** 2013

**Authors:** Jahangir ABDI, Morovat TAHERIKALANI, Kheirolah ASADOLAHI, Mohammad EMANEINI

**Affiliations:** 1 Department of Medical Parasitology, School of Paramedicine, Ilam University of Medical Sciences, Ilam, Iran; 2 Departments of Medical Microbiology, School of Medicine, Ilam University of Medical Sciences, Ilam, Iran; 3 Departments of Epidemiology, School of Medicine, Ilam University of Medical Sciences, Ilam, Iran; 4 Department of Microbiology, School of Medicine, Tehran University of Medical Sciences, Tehran, Iran

**Keywords:** Hydatidosis, Echinococcosis, Seroprevalence, Iran, Stray dogs

## Abstract

**Background:**

Hydatidosis is a zoonotic disease of global prevalence. It causes considerable health problems and economic losses throughout the world, including Iran. The objective of this study was to assess the current status of echinococcosis/hydatidosis in the province of Ilam (western Iran).

**Methods:**

From April to September 2011, 65 stray dogs were collected from urban and rural areas of Ilam City. Parasites were isolated from the dogs and stained with carmine. A taxonomic study was carried out by measuring different parts of helminths. Meat inspection documents from slaughterhouses in Ilam were used to assess the prevalence of hydatidosis during a 3-year period in sheep, cattle, and goats. ELISA test was used to detect the presence of antibodies to hydatidosis in human sera. Clinical records from 2000 to 2010 of either treated or diagnosed patients from public hospitals of this province were reviewed.

**Results:**

The prevalence of *Echinococcus granulosus* infection in stray dogs was 9%. A total of 81,726 animals were assessed for hydatidosis; 2.94% (2403 cases) had liver hydatidosis and 2.34% (1918 cases) had lung hydatidosis. Within a 10-year period, 140 patients (91 females and 49 males) were treated for hydatidosis. Of 1200 human sera, 2.25% (27 patients) were seropositive for hydatidosis.

**Conclusion:**

Hydatidosis is endemic in Ilam Province especially in rural area. The health and economic losses caused by the disease are significant; thus, our efforts need to be focused on the control of this disease.

## Introduction

Hydatidosis is one of the most important parasitic diseases that affect humans and domestic and wild ungulates. The disease is caused by an infection from the taeniid cestode larvae (*Echinococcus*). This parasitic disease has a worldwide prevalence, but it has a particularly high prevalence in Southern Europe, East Africa, Australia, New Zealand, and Latin America (1). Outbreaks of this disease have occurred in Asian countries such as Lebanon, Jordan, Syria, Iraq, Saudi Arabia, and Iran, leading to substantial health problems and economic losses (2–7).

The disease is so deleterious to human health that one of the active programs of the World Health Organization (WHO) that focuses on zoonotic diseases is actively fighting this parasitic disease (8). It is caused by six species of *Echinococcus*; however, four species (*E. granulosus*, *E. multilocularis*, *E. vogeli*, and *E. oligartrus*) pose a significant threat to human health (9, 10).

Numerous studies have focused on echinococcosis and hydatidosis in Iran. The prevalence of *E. granulosus* has been reported as 34.8% among stray dogs in Yasuj (11). In Hamadan City, the seropositivity of human hydatidosis has been reported 3% (12). Moulazade reported that 8.5%, 5.1%, and 7.8% of cow, sheep and goat, respectively, were infected with hydatid cysts in the abattoirs of Jiroft (13). There are reports about the prevalence of the parasite in other countries. For example, in northern Spain, 14% of 721 dogs tested positive for echinococcosis in a coproantigen ELISA test (14). Based on the literature, it is apparent that hydatidosis has a worldwide distribution. There are many researchers who believe that the effects of hydatidosis on human health are more significant than those on the economy; thus, they have focused their efforts on controlling this parasitic disease (15–18). Even though the disease is prevalent in some regions of Iran, the prevalence of hydatidosis in Ilam Province is unknown.

This study was carried out to assess the current status of echinococcosis/hydatidosis and to understand the epidemiology of the disease in Ilam Province, western Iran.

## Materials and Methods

### Study area

Ilam Province has an area of 23,666 square kilometers located in western Iran. It neighbors Khuzestan Province in the south, Lurestan Province in the east, Kermanshah Province in the north, and Iraq in the west, with a 425 km common border. Geographically, most of Ilam Province (especially the north) has hills and mountains. Pastoralism is one of the most important jobs in the area. According to the 2005 census from the Statistical Center of Iran, Ilam Province's population was 563,898; 13% of the population consisted of nomads.

### Collection and examination of stray dogs

From April to September 2011, 65 stray dogs were collected from urban and rural areas of the province. Parasites were isolated from the dogs and kept in 70% ethanol. Two methods were used for the study and diagnosis of the worms: rapid diagnosis and diagnosis following permanent staining. An acid-carmine stain containing 30%, 50%, 75%, 90%, 96%, and 100% alcohol were used for the permanent staining. Xylol was used for clarifying the samples and Kanadabalzam was used for stabilizing the slide. The *Echinococcus* species were determined according to the protocol by Kumaratilake and Thompson (19).

### Examination of slaughtered animals

Meat inspection documents, dating from 2009 to 2012, were used to assess the prevalence of hydatid cysts in sheep, cattle, and goats in a slaughterhouse located in Ilam. This retrospective study assessed 81,726 animals, which included 51,165 sheep, 17,109 goats, and 13,452 cattle. The obtained data was analyzed by SPSS 16.

### Hospitalized patients

Another retrospective study involved estimating the incidence of hydatidosis among patients hospitalized in Ilam Province from 2000 to 2010. All medical records of patients treated for hydatidosis in Ilam were analyzed.

### Human seroprevalence

A total of 1200 human sera from residents in Ilam (700 from women and 500 from men between the ages of 4 to 90) were tested for the presence of antibodies to the hydatid cysts. An *ELISA* using native AgB was conducted to test the presence of antibodies in the serum samples of the patients (5, 20).

## Results

The results showed that 9% of the stray dogs (6 dogs) *tested* positive for E. granulosus at necropsy. Age and sex were not significantly correlated with the prevalence of *E*. *granulosus* (*P* > 0.05) (Table 1). The overall prevalence of hydatidosis in the slaughtered animals was 5.28% (Table 2). The overall human seroprevalence was 2.25% (27 individuals) (Table 3). A total of 140 patients (2.5 per 100,000 individuals) were treated for hydatidosis during the 10-year period (2000-2010). Ninety one of these individuals were female and 49 were male; 59.2% of the infected cases (83 individuals) came from rural areas (Table 3).

**Table 1:** Prevalence of echinococcosis amongst stray dogs in Ilam Province

Sources	Number under study	No of positive cases	Percent of infected cases	Sex of infected cases		Location of infected cases	
				Male	Female	Rural	Urban
**Stray dogs**	65	6	9	4	2	5	1

**Table 2:** Prevalence of hydatidosis amongst animals slaughtered in industrial abattoirs in Ilam Province

Disease condition	Infected Cattle n (%)	Infected Sheep n (%)	Infected Goat n (%)	Total n (%)
**Liver hydatid cyst**	979 (7.27)	1134(2.21)	290(1.69)	2403(2.94)
**Lung hydatid cyst**	361(2.68)	1260(2.46)	297(1.73)	1918(2.34)
**Total**	1340(9.96)	2394(4.67)	587(3.43)	4321(5.28)

**Table 3:** Prevalence of seropositivity for human hydatidosis and incidence of human hydatid cyst surgery in Ilam Province

Sources	Number under study	No. of positive cases	Sex of infected cases		Location of infected cases	
			Male	Female	Rural	Urban
**Human surgery**	A ten year	140	49	91	83	57
**Seropositivity**	1200	27	15	12	19	8

## Discussion

Individuals living in rural areas are more affected than those living in urban areas. This might be attributed to lack of adequate information about the disease, poor human hygiene, and frequent contact with dogs. Compared with other studies conducted in Iran, the prevalence of echinococcosis among stray dogs was low in this study. A study reported an echinococcosis prevalence rate of 17.6% among stray dogs in Kerman (21). In another study, the prevalence of *E. granulosus* was found 22% among stray dogs in Mashhad (22). In Jordan, 14% of dogs (25 dogs) were found infected with *E*. *granulosus* (23). The prevalence of *E. granulosus* has been reported 18.4% in wild carnivores and stray dogs of northern Tunisia, Spain (24).-Therefore, the prevalence of *E*. *granulosus* in these studies was higher than that of the present study (9%). This difference could be due to recent droughts in Ilam Province. Based on the literature, *E. granulosus* has a worldwide distribution and adequate actions have not been undertaken to reduce its prevalence. Many reports exist on animal hydatidosis, caused by the larvae of *E. granulosus*. The hydatidosis infection rate has been reported 4.0% in sheep, 3.6% in goats, 11.4% in cattle, and 8.8% in camels in two slaughterhouses in North Jordan (25). Hydatid cysts were detected in 35.2% (233/661) of camels slaughtered in different regions of Iran (26). In the study on slaughtered animals from Qom Province (central part of Iran), 9.3% of sheep, 2% of goats, and 3.5% of cattle were infected with hydatid cysts (27).

A comparison between the present study and the above studies reveals that the infection rate of echinococcosis/hydatidosis in Ilam is different to that present in the rest of the world, suggesting a different distribution pattern of the parasites.

Humans can also become infected with hydatid cysts. A human seroprevalence of 2.4% and 9.5% have been reported in Jordan and China, respectively (28, 29). Aflaki et al. (4) reported that 1.2% of individuals living in Ilam were seropositive for hydatidosis using the dot-ELISA. Although the tests used in the two studies were different (i.e., ELISA in the present study vs. dot-ELISA in that study) the results still reveal that in recent years the rate of hydatid cysts has increased in Ilam Province. A study conducted in the Meshkin-shahr district (30) revealed that 1.79% of humans tested positive for hydatidosis, according to an ELISA test; this result was similar to that obtained in the present study. The seropositivity of human hydatidosis in the IX Region of la Araucania, Chile was 38.5 per 100,000 individuals (31). According to the hospital medical records that we obtained, 140 patients (2.5 per 100,000) were treated for hydatid cysts in a 10-year period. Individuals living in rural areas (e.g., pastoralists and shepherds) were more prone to be infected with hydatid cysts than those living in urban communities. The majority of the infected individuals had hydatid cysts localized in the liver.

## Conclusion

Apparently echinococcosis is endemic in Ilam Province. It is a serious health problem, and dogs play an important role as a reservoir and definitive host. There is no information on disease transmission among people. In addition to its deleterious effect on human health, there are considerable economic losses resulting from this disease. Currently, the control of stray dogs in urban areas and the treatment with medication of herding dogs in rural areas, improves public health. It is crucial to control and prevent parasitic diseases in animals and to find possible sources of infection (e.g., water, soil, vegetables, etc.).
